# Tourism experiences and the lower risk of mortality in the Chinese elderly: a national cohort study

**DOI:** 10.1186/s12889-021-11099-8

**Published:** 2021-05-27

**Authors:** Min Du, Liyuan Tao, Min Liu, Jue Liu

**Affiliations:** 1 Department of Epidemiology and Biostatistics, School of Public Health, Peking University, No.38, Xueyuan Road, Haidian District, Beijing, 100191 China; 2 Research Center of Clinical Epidemiology, Peking University Third Hospital, No.49 Huayuan North Road, Haidian District, Beijing, 100083 China; 3 Institute for Global Health and Development, Peking University, Beijing, 100871 China; 4 Key Laboratory of Reproductive Health, National Health Commission of the People’s Republic of China, Beijing, 100083 China

**Keywords:** Tourism, Mortality, Chinese, Older, Cohort

## Abstract

**Background:**

Cohort studies about the effects of tourism experiences on the risk of death among Chinese older adults are still lacking. We aimed to examine the association between tourism experiences and mortality in Chinese aged 65 or above.

**Methods:**

We included 9520 participants aged 65 years or above from the Chinese Longitudinal Healthy Longevity Survey at baseline in 23 provinces in 2011. They were followed up in 2014 and 2018. Cox proportional hazards models were used to assess the association between tourism experiences and the risk of death.

**Results:**

Among 9520 participants, 7.85% had at least one tourism experience outside of their local city/county during the past 2 years. During 35,994.26 person-years of follow-up, in total 4635 deaths were observed. The crude rate of death was greater in participants who had no tourism experience than in older travelers (incidence rate: 13.70 versus 5.24 per 100 person-years). Elderly travelers had a significantly lower risk for all-cause mortality (crude hazard ratio: 0.38, 95% CI: 0.33–0.44) compared with non-travelers. After adjustment for all covariates, the risk of all-cause mortality was 27% lower among those with at least one tourism experience than among non-travelers (adjusted hazard ratio: 0.73, 95% CI: 0.62–0.85). Subgroup analysis showed that the associations between tourism and the decreased risk of mortality were stable.

**Conclusions:**

Tourism decreases the risk of mortality in the Chinese elderly. Tourism should be considered as a modifiable lifestyle factor and an effective way to reduce mortality and promote longevity and healthy aging.

**Supplementary Information:**

The online version contains supplementary material available at 10.1186/s12889-021-11099-8.

## Background

Aging has become one of the great challenges worldwide [1–4]. According to the 2019 China Statistical Yearbook, there were 176 million people aged 65 or above, which accounted for 12.6% of the whole population in China [3]. Furthermore, it is estimated that there will be 400 million Chinese citizens aged ≥65, 150 million of whom will be aged ≥80, by 2050 [4]. The issue of aging implies broad global health implications, including mortality [5]. In the context of the disease burden from rapid population aging, behavior interventions such as reducing smoking [6], improving diet [7, 8], and appropriate physical activity have played an increasingly important role in shaping health among older adults.

Tourism is one type of physical activity. A previous meta-analysis has shown that physical activity can reduce the risk of death [9, 10], and sedentary behavior [11] can increase the risk of death in the general population. Previous studies in Brazil [3], Japan [12], Spain [13], and Korea [14] also reported that physical activity can reduce the risk of death among elderly, and absence of outdoor activities can increase the risk of death among older people [15]. However, studies reporting on the association between tourism and death among the general population or the elderly are scarce. However, it has been found that tourism is associated with the health situation of older people. One previous study reported that “forest bathing” (*shinrin-yoku*) trips have a positive effect on health among older patients with chronic obstructive pulmonary disease by reducing inflammation and stress levels [16]. Another cross-sectional study found that tourism was positively associated with better self-rated health among Chinese people [17]. Chang et al. demonstrated that participation in domestic nature-based tourism served as a tonic to improve autonomic nervous system function [18]. Additionally, tourism has been reported to be beneficial for the psychological state, including wellbeing perception [19], perceived accessibility, and life satisfaction [20].

In fact, with the aging population, older people are more active in travel participation than previous generations [21, 22]. Although there is evidence with respect to the association between tourism experiences and some health indices (including self-rated health, autonomic nervous system function, and psychological situation) among older people, it remains unclear whether tourism experiences have a direct effect on death after controlling for related confounding factors, including health status, exercise, and other leisure activities [17]. The association between tourism experiences and death among elderly is an emerging research field [21]. The Chinese Longitudinal Healthy Longevity Survey (CLHLS) was a nationally representative population-based survey that included 23 out of 31 provinces in China. This survey was utilized broadly in the aging research among Chinese elderly because of the complete information of elderly, such as demographics, lifestyle, and health status [23]. Therefore, in this study, we examined the association between tourism experiences and mortality in Chinese adults aged 65 years or above by using the data from the CLHLS, which can provide evidence on the links between tourism experiences and death among the elderly.

## Methods

### Participants

We used data from the CLHLS, a prospective nationwide cohort study that covered about 85% of the total population from a randomly selected half of the counties and cities in 23 of 31 provinces in China. A targeted random-sample design was adopted to ensure representativeness. All of the centenarians of the sampled counties and cities agreed voluntarily to participate in the study. This study was established in 1998, with subsequent follow-up and recruitment of new participants in 2000, 2002, 2005, 2008, 2011, 2014, and 2018. This design serves well to provide information on the health status and quality of life among older people aged 65 or above [23].

The present analysis included data from the 2011 wave of the CLHLS (at baseline), which included the question “How many times did you have any tourism experience beyond your home county/city within the past two years?” The follow-up survey was conducted in 2014 and 2018. The CLHLS was approved by the Ethical Review Committee of Peking University (IRB00001052–13074). All of the participants signed informed consent at the time of participation. The research has been performed in accordance with the Declaration of Helsinki.

**Fig. 1:**
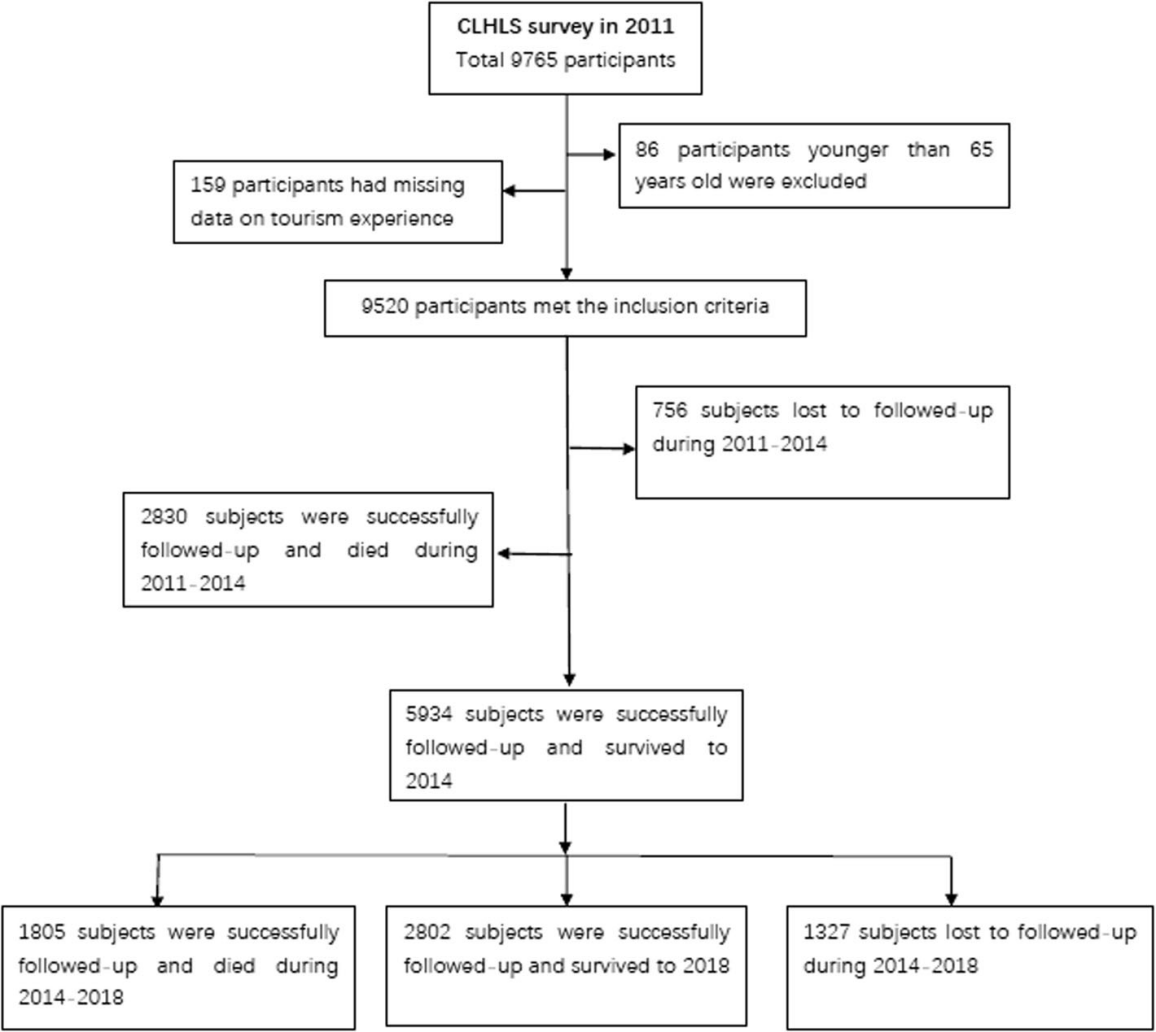
Flowchart of the inclusion of participants

The 2011 wave included 9765 Chinese elderly individuals. We excluded 86 participants which were younger than 65 years old, and 159 participants for whom data on tourism experiences were missing. For the analysis of the association between tourism experience and all-cause mortality, in total 9520 participants were included. Among these, 756 participants were lost to follow-up in 2014 and 1327 participants were lost to follow-up in 2018. Figure 1 shows the selection process of research participants in this study.

### Assessment of tourism experiences

Participants’ tourism experiences were obtained from the questionnaire answered at baseline by asking the participants the following question: “How many times did you have any tourism experience beyond your home county/city within the past two years?” Because only 4.5% of participants had two or more tourism experiences, according to the previous studies, participants were classified into two groups: those who had at least one tourism experience and those who had not [17].

### Covariates

Trained investigators collected information, including basic demographic characteristics, lifestyle, health status, and socioeconomic status, using a standardized questionnaire. All of the surveys were face-to-face interviews conducted at the participants’ homes. If participants were illiterate, investigators helped them to complete the questionnaire. Details of the study design have been described elsewhere, and the quality of the data was reported to be generally good [24].

Basic demographic characteristics included age (< 85/≥ 85), sex (male/female), education (no school/1 year or more), residence (urban/rural), marital status (unmarried/married/divorced or widowed), and living pattern (living with family members/living alone or in an institution).

Lifestyle characteristics included smoking status (non-smoker/smoker), drinking status (non-drinker/drinker), regular exercise (yes/no), leisure activities, and dietary diversity score (DDS, good/poor). According to a study by Gu et al. [17], the frequency of involvement in leisure activities and doing regular exercise were considered competing activities that may confound the effect of tourism participation on health, so we included regular exercise and leisure activities. Leisure activities were measured by asking about the frequencies of eight dummy leisure activities, including housework, gardening, outdoor activities, raising poultry or pets, reading, playing cards/Mahjong, listening to the radio/watching television, and participating in organized social activities. The answer of each leisure activity was categorized as 0 (never) or 1 (sometimes or almost). We summed the scores (ranging from 0 to 8) to calculate the frequency of leisure activities [17]. The DDS was categorized according to the recommendations by the Food and Agriculture Organization of the United Nations and previous research [25].

Health status was measured on the basis of body mass index (BMI) (underweight/normal/overweight/obese), depression (yes/no), history of chronic diseases (hypertension, diabetes, heart diseases, and stroke; yes/no/unknown), history of cancer (yes/no/unknown), history of arthritis (yes/no/unknown), cognitive impairment (yes/no), activities of daily living (ADL in disability; yes/no), and toothache or pain in the jaw joint more than once during the past 6 months (yes/no). In accordance with the World Health Organization cutoff values, BMI was categorized as underweight (< 18.5 kg/m^2^), normal (18.5–24.9 kg/m^2^), overweight (25–29.9 kg/m^2^), or obese (≥ 30 kg/m^2^). Depression was assessed by the following question: “Have you felt sad, blue, or depressed for two weeks or more in the last 12 months?” Cognitive function was measured by using the Chinese version of the Mini-Mental State Examination (MMSE), which consists of 11 questions covering orientation, registration, attention, calculation, recall, and language abilities [26]. Several items of the MMSE were adapted to the Chinese cultural context with good validity and reliability [27]. The total MMSE scores ranged from 0 to 30; CLHLS participants who scored less than 18 in the Chinese version of the MMSE were classified as having cognitive impairment, whereas participants with a score of 18 or higher were classified as having no cognitive impairment [28, 29]. ADL refers to basic personal care tasks of everyday life. In this study, ADL in disability was defined as self-reported difficulty with any of the following ADL items [30]: dressing, eating, bathing, continence, toileting, cleaning, and indoor movement.

Socioeconomic status (SES) was assessed by two indices, including childhood SES and adult SES. We evaluated childhood SES by the following question: “Did you often go to bed hungry as a child?” (yes/no/unknown). Adult SES was evaluated by the following question: “How do you rate your economic status compared with other local people?” Participants who answered “very rich” or “rich” were classified as good adult SES, and participants who answered “so-so,” “poor,” or “very poor” were classified as poor adult SES.

### Data analysis

Baseline characteristics of the study population are presented as the mean ± standard deviation (SD) for continuous variables or as percentages for categorical variables. Time to death (event = 1) was defined as the period between the baseline survey and death. This study included two follow-up surveys, in 2014 and 2018. For participants who died before the 2014 follow-up, family members were contacted to investigate the date of death based on the medical records. For participants who died between the 2014 and 2018 follow-ups, the date of death was investigated in the same way. Censoring (event = 0) was performed for surviving participants or those lost to follow-up in 2014. The censoring time was calculated from baseline to the survey of 2014/2018, which was updated. Survival was estimated by the Kaplan–Meier method (Fig. S1), and the difference in survival was evaluated with a stratified log-rank test (*P* < 0.0001). Cox proportional hazards models were used to assess the association of tourism experiences with all-cause mortality. The proportional hazard assumption was evaluated by tests based on Schoenfeld residuals [31], which showed no violation of this assumption in our analyses (*P* = 0.17). We performed a sensitivity analysis by fitting different models to examine the robustness of the estimation. Model 1 was a univariate model without adjustment for any confounders. We adjusted for basic demographic characteristics, including age, sex, education, residence, marital status, and living pattern, in model 2. We adjusted for all of the covariates in model 3 by adding smoking status, drinking status, regular exercise, leisure activities, dietary diversity, BMI, depression, cognitive impairment, ADL in disability, self-reported chronic diseases including hypertension, diabetes, heart diseases, and stroke, history of cancer, history of arthritis, toothache or pain in the jaw joint more than once during the past 6 months, childhood SES, and adult SES. The missing data of every covariates used in this study was all less than 5%. Therefore, we did not further treat missing values (such as multiple imputation). In the models, if there was missing data, the cases were not included in the analysis. We calculated crude incidence rates (IRs) (per 100 person-years) of death across categories of tourism experiences. Our results are presented as pooled hazard ratios (HRs) or β-values with 95% confidence intervals (CIs).

Additionally, a stratified analysis was performed by age, sex, education, residence, marital status, living pattern, smoking status, drinking status, regular exercise, leisure activities, dietary diversity, BMI, depression, cognitive impairment, ADL in disability, self-reported chronic diseases, history of cancer, history of arthritis, and toothache or pain in the jaw joint more than once during the past 6 months, and the significance of the interaction was tested by including a two-way interaction term in the final model.

In order to test the robustness of the results, we did additional analysis as followings: (1) There were 756 participants who never responded to the follow-up, so we compared the characteristics between 756 participants and the other 8764 participants, which showed that differences of most characteristics were insignificant (Table S1). (2) Cox models were performed for the 8764 participants who responded at least once to follow-up (in 2014 or 2018, excluding the 756 participants who never responded) (Table S2). (3) For the 9520 participants at baseline, Cox model A, which was adjusted for all of the covariates except regular exercise and leisure activities, and model B, which was adjusted for all of the covariates after multiple imputation of missing values, were generated (Table S3).

*P*-values below 0.05 were considered to indicate statistical significance. All of the analyses were performed with SPSS 26.0 and Stata 16.0.

## Results

### Basic characteristics of the participants

Characteristics of the 9520 participants at baseline are shown in Table 1. The mean age was 85.95 (±11.21) years, and 44.78% of participants were men. Overall, 7.85% (747/9520) of participants had at least one tourism experience outside of their local city/county during the previous 2 years at baseline.

**Table 1:** Characteristics of the study participants according to tourist experiences at baseline

Characteristics	N	Tourism experiences		***t***/χ^**2**^	***P***
		Mean ± SD or ***n*** (%)			
		0 times within the past two years	≥1 times within the past two years		
**Total**		8773 (92.15)	747 (7.85)		
**Basic demographic characteristics**					
Age (years)				248.867	< 0.0001
< 85	4417	3864 (87.48)	553 (12.52)		
≥ 85	5103	4909 (96.20)	194 (3.80)		
Sex				25.202	< 0.0001
Male	4263	3863 (90.62)	400 (9.38)		
Female	5257	4910 (93.40)	347 (6.60)		
Education				209.587	< 0.0001
No school	5535	5286 (95.50)	249 (4.50)		
1 year or more	3944	3446 (87.37)	498 (12.63)		
Residence				93.101	< 0.0001
Urban	4544	4061 (89.37)	483 (10.63)		
Rural	4976	4712 (94.69)	264 (5.31)		
Marital status				135.646	< 0.0001
Unmarried	98	93 (94.90)	5 (5.10)		
Married	3586	3157 (88.04)	429 (11.96)		
Divorced or widowed	5806	5496 (94.66)	310 (5.34)		
Living pattern				1.291	0.256
Living with family members	7647	7033 (91.97)	614 (8.03)		
Living alone/in an institution	1812	1681 (92.77)	131 (7.23)		
**Lifestyle characteristics**					
Smoking status				21.957	< 0.0001
Non-smoker	6254	5819 (93.04)	435 (6.96)		
Smoker	3218	2906 (90.30)	312 (9.70)		
Drinking status				23.601	< 0.0001
Non-drinker	6467	6018 (93.06)	449 (6.94)		
Drinker	2956	2665 (90.16)	291 (9.84)		
Regular exercise				205.746	< 0.0001
Yes	4302	3778 (87.82)	524 (12.18)		
No	5101	4887 (95.80)	214 (4.20)		
Leisure activities	9492	2.62 (±1.87)	4.40 (±1.77)	−26.274	< 0.0001
Dietary diversity score				177.506	< 0.0001
Poor	4868	4661 (95.75)	207 (4.25)		
Good	4648	4109 (88.40)	539 (11.60)		
**Health status**					
Body mass index (kg/m^2^)				70.359	< 0.0001
Underweight (< 18.5)	2379	2268 (95.33)	111 (4.67)		
Normal (18.5–24.9)	5398	4933 (91.39)	465 (8.61)		
Overweight (25–29.9)	1062	931 (87.66)	131 (12.34)		
Obese (≥30)	288	254 (88.19)	34 (11.81)		
Depression				89.841	< 0.0001
Yes	1200	1102 (91.83)	98 (8.17)		
No	6725	6109 (90.84)	616 (9.16)		
Unknown	1595	1562 (97.93)	33 (2.07)		
Hypertension				15.416	< 0.0001
Yes	2695	2444 (90.69)	251 (9.31)		
No	6414	5937 (92.56)	477 (7.44)		
Unknown	411	392 (95.38)	19 (4.62)		
Diabetes				34.445	< 0.0001
Yes	393	333 (84.73)	60 (15.27)		
No	8629	7969 (92.35)	660 (7.65)		
Unknown	498	471 (94.58)	27 (5.42)		
Heart diseases				15.729	< 0.0001
Yes	1155	1031 (89.26)	124 (10.74)		
No	7892	7300 (92.50)	592 (7.50)		
Unknown	473	442 (93.45)	31 (6.55)		
Stroke				2.1	0.35
Yes	783	732 (93.49)	51 (6.51)		
No	8301	7640 (92.04)	661 (7.96)		
Unknown	436	401 (91.97)	35 (8.03)		
Cancer				9.012	0.011
Yes	81	68 (83.95)	13 (16.05)		
No	8896	8197(92.14)	699 (7.86)		
Unknown	543	508 (93.55)	35 (6.45)		
Arthritis				6.986	0.030
Yes	1319	1192 (90.37)	127 (9.63)		
No	7781	7190(92.40)	591 (7.60)		
Unknown	420	391 (93.10)	29 (6.90)		
Cognitive impairment				154.251	< 0.0001
Yes	2349	2305 (98.13)	44 (1.87)		
No	7160	6457 (90.18)	703 (9.82)		
ADL in disability				103.425	< 0.0001
Yes	2473	2395 (96.85)	78 (3.15)		
No	6825	6171 (90.42)	654 (9.58)		
Toothache or pain in the jaw joint more than once during the past 6 months				40.357	< 0.0001
Yes	1654	1461 (88.33)	193 (11.67)		
No	7596	7062 (92.97)	534 (7.03)		
**Socioeconomic status**					
Childhood SES				33.618	< 0.0001
Yes	6315	5862 (92.83)	453 (7.17)		
No	2024	1804 (89.13)	220 (10.87)		
Unknown	1181	1107 (93.73)	74 (6.27)		
Adult SES				94.262	< 0.0001
Good	1619	1395 (86.16)	224 (13.84)		
Poor	7800	7279 (93.32)	521 (6.68)		

Notes: Missing data: Basic demographic characteristics: education 41 (0.43%), marital status 30 (0.32%), and living pattern 61 (0.64%); Lifestyle characteristics: smoking status 48 (0.50%), drinking status 97 (1.02%), regular exercise 117 (1.23%), leisure activities 28 (0.29%), DDS 4 (0.04%); health status: BMI 393 (4.13%), cognitive impairment 11 (0.12%), ADL in disability 222 (2.33%), toothache or pain in the jaw joint more than once during the past 6 months 270 (2.84%); socioeconomic status: adult SES 101 (1.06%)

Participants who were male, aged < 85 years, literate, married, citizen, smoker, drinker, overweight, and not depressed, participants who performed more leisure activities or did regular exercise, and participants with good dietary diversity, chronic disease (including hypertension, diabetes, and heart diseases), cancer, arthritis, toothache or pain in the jaw joint more than once during the past 6 months, and good childhood SES and good adult SES and without cognitive impairment and disability were more likely to have had a tourism experience during the past 2 years (*P* < 0.05, Table 1). However, tourism experiences were not associated with living patterns and history of stroke (*P* > 0.05).

### Association of tourism experiences with all-cause mortality

During 35,994.26 person-years of follow-up (median survival time: 2.88 years overall; 2.86 years for the group with zero tourism experiences within the past 2 years; 5.34 years for the group with ≥1 tourism experience within the past 2 years), a total of 4635 deaths were observed, including 4452 participants who had no tourism experience and 183 older travelers (IR: 13.70 versus 5.24 per 100 person-years). Overall, the crude rate of death was higher in participants who had tourism experience than in older travelers (Table 2). In the unadjusted analysis, older travelers had a lower risk for all-cause mortality (crude HR [cHR]: 0.38, 95% CI: 0.33–0.44). After adjusting for basic demographic characteristics including age, sex, education, residence, marital status, and living pattern, the association remained significant (adjusted HR [aHR]: 0.57, 95% CI: 0.49–0.66). In multivariable models, after adjustment for all covariates, compared to the older people who had no tourism experience, the risk of all-cause mortality was 27% lower among older travelers (aHR: 0.73, 95% CI: 0.62–0.85) (Table 2).

**Table 2:** Association of tourism experiences with mortality in the univariate and multivariable models

Tourist experience	Number of events/incidence rate (per 100 person-years)	Model 1		Model 2		Model 3	
		cHR (95% CI)	*P*	aHR (95% CI)	*P*	aHR (95% CI)	*P*
0 times within the past 2 years	4452/13.70	1 (reference)		1 (reference)		1 (reference)	
≥ 1 times within the past 2 years	183/5.24	0.38 (0.33–0.44)	< 0.0001	0.57 (0.49–0.66)	< 0.0001	0.73 (0.62–0.85)	< 0.0001

Notes: Model 1 is a univariate model. In model 2, we adjusted for basic demographic characteristics, including age, sex, education, residence, marital status, and living pattern. In model 3, we adjusted for all covariates by adding smoking status, drinking status, regular exercise, leisure activities, dietary diversity, BMI, depression, cognitive impairment, ADL in disability, history of chronic disease (hypertension, diabetes, heart diseases, and stroke), history of cancer, history of arthritis, toothache or pain in the jaw joint more than once during the past 6 months, childhood SES, and adult SES. cHR, crude hazard ratio; aHR, adjusted hazard ratio

### Subgroup analysis

In the subgroup analysis, the associations between tourism and the risk of mortality were stable. We stratified the analysis by age, sex, education, residence, marital status, living pattern, smoking status, drinking status, regular exercise, leisure activities, dietary diversity, BMI, depression, cognitive impairment, ADL in disability, self-reported chronic diseases, history of cancer, history of arthritis, and toothache or pain in the jaw joint more than once during the past 6 months in the multivariable model. Significant group differences in smoking status were found on the basis of the association of tourism experiences with mortality in the multivariable-adjusted model (*P*-values for the interaction < 0.05). The risk of mortality was lower among participants who never smoked (aHR: 0.64, 95% CI: 0.51–0.80). No interaction was observed within the other groups (Table 3).

**Table 3:** Subgroup analysis for the association of tourism experiences with mortality

Subgroup	≥1 times within the past 2 years	
	Adjusted HR (95% CI)	*P* for interaction
All	0.73 (0.62–0.85)	
**Basic demographic characteristics**		
Age (years)		0.888
< 85	0.80 (0.64–1.01)	
≥ 85	0.70 (0.56–0.87)	
Sex		0.544
Male	0.75 (0.61–0.93)	
Female	0.69 (0.53–0.88)	
Education		0.539
No school	0.68 (0.52–0.87)	
1 year or more	0.76 (0.62–0.94)	
Residence		0.519
Urban	0.66 (0.54–0.81)	
Rural	0.85 (0.65–1.09)	
Marital status		0.806
Unmarried	0.47 (0.04–6.34)	
Married	0.71 (0.55–0.92)	
Divorced or widowed	0.74 (0.60–0.91)	
Living pattern		0.180
Living with family members	0.76 (0.64–0.90)	
Living alone/in an institution	0.59 (0.38–0.89)	
**Lifestyle characteristics**		
Smoking status		0.027
Non-smoker	0.64 (0.51–0.80)	
Smoker	0.82 (0.65–1.04)	
Drinking status		0.327
Non-drinker	0.79 (0.64–0.97)	
Drinker	0.66 (0.51–0.86)	
Regular exercise		0.643
Yes	0.73 (0.60–0.89)	
No	0.70 (0.53–0.94)	
Leisure activities (median)		0.974
0–3	0.73 (0.58–0.92)	
4–8	0.70 (0.56–0.88)	
Dietary diversity score		0.643
Poor	0.75 (0.57–0.99)	
Good	0.73 (0.60–0.88)	
**Health status**		
Body mass index (kg/m^2^)		0.482
Underweight (< 18.5)	0.72 (0.52–0.99)	
Normal (18.5–24.9)	0.69 (0.56–0.85)	
Overweight (25–29.9)	0.66 (0.70–1.77)	
Obese (≥30)	0.65 (0.23–1.87)	
Depression		0.442
Yes	0.62 (0.40–0.96)	
No	0.75 (0.63–0.90)	
Unknown	0.59 (0.29–1.20)	
Hypertension		0.633
Yes	0.78 (0.58–1.05)	
No	0.72 (0.60–0.88)	
Unknown	0.41 (0.14–1.23)	
Diabetes		0.666
Yes	0.80 (0.45–1.42)	
No	0.72 (0.60–0.85)	
Unknown	0.60 (0.24–1.53)	
Heart diseases		0.753
Yes	0.78(0.51–1.19)	
No	0.74 (0.62–0.88)	
Unknown	0.51 (0.20–1.28)	
Stroke		0.223
Yes	1.64 (0.97–2.76)	
No	0.68 (0.57–0.81)	
Unknown	0.61 (0.29–1.31)	
Cancer		0.724
Yes	0.05 (0.01–0.57)	
No	0.72 (0.61–0.85)	
Unknown	0.72 (0.34–1.53)	
Arthritis		0.670
Yes	0.89 (0.60–1.33)	
No	0.70 (0.58–0.84)	
Unknown	0.79 (0.34–1.81)	
Cognitive impairment		0.448
Yes	0.87 (0.57–1.32)	
No	0.72 (0.60–0.85)	
ADL in disability		0.239
Yes	0.84 (0.60–1.19)	
No	0.71 (0.59–0.85)	
Toothache or pain in the jaw joint more than once during the past 6 months		0.674
Yes	0.58 (0.41–0.84)	
No	0.77 (0.65–0.93)	
**Socioeconomic status**		
Childhood SES		0.369
Yes	0.74 (0.61–0.91)	
No	0.76 (0.56–1.04)	
Unknown	0.47 (0.24–0.94)	
Adult SES		0.315
Good	0.86 (0.64–1.15)	
Poor	0.68 (0.56–0.83)	

Notes: We adjusted for all covariates, including age, sex, education, residence, marital status, living pattern, smoking status, drinking status, regular exercise, leisure activities, dietary diversity, BMI, depression, cognitive impairment, ADL in disability, history of chronic disease (hypertension, diabetes, heart diseases, and stroke), history of cancer, history of arthritis, toothache or pain in the jaw joint more than once during the past 6 months, childhood SES, and adult SES. cHR, crude hazard ratio; aHR, adjusted hazard ratio

## Discussion

To our knowledge, this is the first nationwide cohort study that examined the association of tourism experiences with all-cause deaths among Chinese older people aged ≥65 years. In this prospective cohort study, we found that elderly people who had at least one tourism experience within the past 2 years had a significantly decreased risk of all-cause death, after adjustment for age, sex, education, residence, marital status, living pattern, smoking status, drinking status, regular exercise, leisure activities, dietary diversity, BMI, depression, cognitive impairment, ADL in disability, history of chronic disease (hypertension, diabetes, heart diseases, and stroke), history of cancer, history of arthritis, toothache or pain in the jaw joint more than once during the past 6 months, childhood SES, and adult SES.

Although there was no study that explored the relationship between tourism experiences and mortality among elderly, our findings still echoed some findings from the limited existing research regarding the effect of tourism on physiology and health among older people. Gu et al. reported that after controlling for confounders such as demographic variables, SES, and health behaviors, individuals who had one or more tourism experiences in the past 2 years were 20% less likely to report poor health (odds ratio: 0.80, *P* < 0.01) among Chinese older people in a panel study [17]. Chang et al. found that participation in domestic nature-based tourism served as a tonic to improve autonomic nervous system function (β = 0.20, *P* < 0.01) [18]. Considering tourism is likely to be a type of outdoor activity, studies which explored the effects of participation in similar activities on the risk of death showed similar results. Sufficient physical activity was reported to be a protective factor for death among elderly [3, 12–14], and insufficient activity can increase the risk of death among older people [15]. Moreover, it is assumed that people with more tourism experiences may have had more exposure to nature. A meta-analysis showed that exposure to greenery is associated with a reduced risk of all-cause mortality (HR: 0.99, 95% CI: 0.97–1.00) [32]. Jia et al. found that “forest bathing” trips have beneficial effects on chronic obstructive pulmonary disease in older patients by decreasing the serum levels of interferon-γ, interleukin-6, and interleukin-8 [16]. Previous limited studies also explored the association of tourism with the psychological state, including perceived wellbeing [19], perceived accessibility, and life satisfaction [20]. Our study was conducted in a large area including 23 research locations in 23 provinces in mainland China, and we corrected for basic demographic characteristics, lifestyle, health-related factors, and socioeconomic status, which could be related to tourism or mortality. We found that after adjustment for all of the covariates, compared to elderly without travel experience, older travelers had a 27% lower risk of death. That is, given the biopsychosocial impact and the comfortable options for older people, tourism appears particularly well suited for older people to reduce mortality. We found that older travelers had a lower risk of death than the entire elderly population, irrespective of regular exercise and leisure activities. This finding may suggest that tourism experience has a strong protective effect on mortality among elderly, independent from exercise and leisure activities.

The specific potential biological mechanism between tourism and all-cause mortality may be related with the effect of physical activity on the human body. Physical activity may stimulate anti-tumor and antioxidant defense systems [14]. In addition, physical activity has been reported to be associated with lower mortality among older people with low serum lipid profiles [33], high insulin sensitivity [34], and reduced muscle inflammation [35]. Of note, biological mechanisms linking tourism and all-cause mortality need to be further explored and verified.

In the present study, 7.85% of participants had one or more tourism experiences during the past 2 years, which was similar to previous studies [17]. Besides, we found that a wide array of factors distinguishes tourists from non-tourists among the older Chinese population, including demographic characteristics (such as sex, age, and education), lifestyle (such as smoking, drinking, and leisure activities), socioeconomic status (childhood SES and adult SES), and health status (such as BMI, chronic disease history, and depression). Health conditions arose as the most frequently indicated barrier for participation in tourism among older Polish people [36]. We found that participants with depression and cognitive impairment were less likely to travel, suggesting that mental health may also be a barrier for physical activity among older people.

### Strengths and limitations

A major strength of this national cohort study is our estimation of the impact of tourism experience on all-cause mortality among older people in China, the first such study of its kind. However, we wish to highlight several limitations. First, we only included participants from mainland China, so the results may not be generalizable to populations form other nations, because different habits and customs exist in different countries. Second, the specific tourism type, such as nature/city trips, time spent traveling, and the transportation mode used, were not collected, so we could not explore deeply about the effect of trips on mortality deeply. Third, SES was obtained through relatively simple questions and need to be improved by multiple indexes or questions in the future. Fourth, although we controlled health-related factors, including chronic disease history, cognition, history of cancer, and arthritis, as much as possible, it is impossible to completely control all health-related factors, which may have had a confounding effect on our results. Finally, the specific cause of death was not recorded in the CLHLS study, so we could not explore the association of tourism with specific causes of death.

## Conclusions

In this nationwide prospective cohort study, tourism experience was associated with reduced mortality in the Chinese elderly population. Our findings suggested that tourism should be considered as a modifiable lifestyle factor for the older people to reduce mortality. Additionally, tourism is an effective way to promote longevity and healthy aging.

## Supplementary Information

**Additional file 1: Figure S1.** Overall Survival among all 9750 Participants. **Table S1.** Comparison of characteristics between 756 participants who never responded to the follow-up and others among all 9520 participants at baseline. **Table S2.** Association of tourism experiences with mortality in the univariate and multivariable models among 8764 participants who responded at least once to the follow-up 2014 or 2018. **Table S3.** Association of tourism experiences with mortality in the multivariable model.

## Data Availability

Data are from the Chinese Longitudinal Healthy Longevity Survey 2011–2018 which is a public, open access repository (https://opendata.pku.edu.cn).

## Abbreviations

ADL: Activities of daily living
BMI: Body mass index
CLHLS: Chinese Longitudinal Healthy Longevity Survey
DDS: Dietary diversity score
HR: Hazard ratios
IR: Incidence rates
MMSE: Mini-Mental State Examination
SDs: Standard deviations
