# Supplementary material for: Tourism experiences and the lower risk of mortality in the Chinese elderly: a national cohort study
Source: BMC Public Health. 2021 May 27;21:996. doi: 10.1186/s12889-021-11099-8 (PMC8157616; doi:10.1186/s12889-021-11099-8)
Supplement: Supplementary file 1 — Additional file 1: Figure S1. Overall Survival among all 9750 Participants. Table S1. Comparison of characteristics between 756 participants who never responded to the follow-up and others among all 9520 participants at baseline. Table S2. Association of tourism experiences with mortality in the univariate and multivariable models among 8764 participants who responded at least once to the follow-up 2014 or 2018. Table S3. Association of tourism experiences with mortality in the multivariable model. [file 12889_2021_11099_MOESM1_ESM.docx]

Supplemental materials：


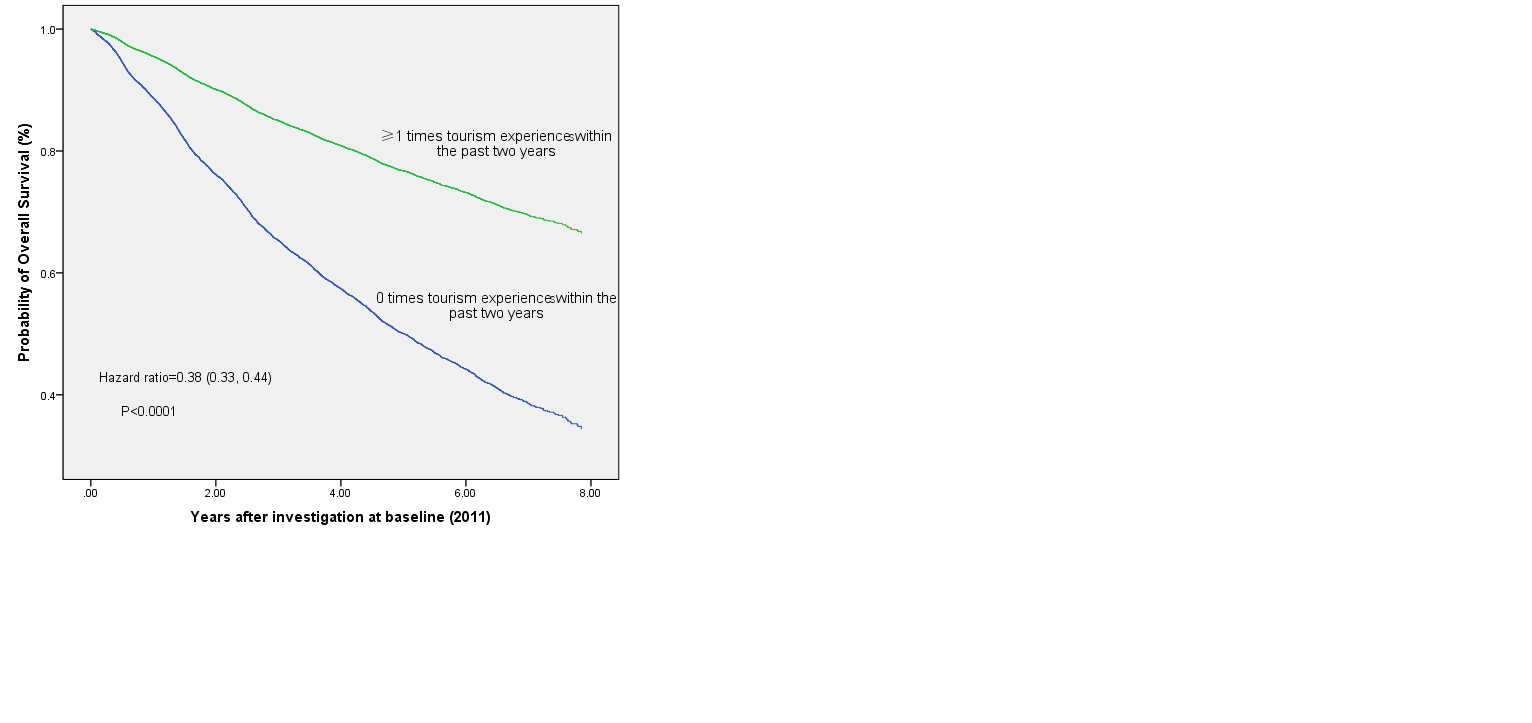


**Figure S1 Overall Survival among all 9750 Participants.** The hazard ratio indicates the risk of death in the ≥1 tourism experiences within the past two years group as 0 times tourism experiences within the past two years group; 95% confidence interval is shown in parentheses. P value was calculated with the use of the stratified log-rank test.

**Table S1 Comparison of characteristics between 756 participants who never responded to the follow-up and others among all 9520 participants at baseline**

| **Characteristics** | **All included Participants** | | | **t/ χ2** | ***P*** |
| --- | --- | --- | --- | --- | --- |
|  | **Mean ± SD or N (%)** | | |  |  |
|  | **All** | **Who never responded to the follow-up** | **Others** |  |  |
| **Total** |  | 756(7.94) | 8764 (92.06) |  |  |
| **Basic demographic characteristics** |  |  |  |  |  |
| Age (years)<85 | 4417 | 349 (46.16) | 4068 (46.42) | 0.018 | 0.893 |
| Male | 4263 | 334 (44.18) | 3929 (44.83) | 0.119 | 0.73 |
| No school | 5535 | 384 (51.34) | 5151 (59.00) | 16.638 | <0.0001 |
| Rural | 4976 | 411 (54.37) | 4565 (52.09) | 1.446 | 0.229 |
| Divorced or Widowed | 5806 | 460 (60.93) | 5346 (61.20) | 0.025 | 0.987 |
| Living with family members | 7647 | 577 (76.73) | 7070 (81.20) | 8.932 | 0.003 |
| **Lifestyle characteristics** |  |  |  |  |  |
| Non-smoker | 6254 | 509 (67.69) | 5745 (65.88) | 1.003 | 0.316 |
| Non-drinker | 6467 | 559 (74.63) | 5908 (68.11) | 13.619 | <0.0001 |
| Regular exercise | 4302 | 341 (46.14) | 3961 (45.72) | 0.05 | 0.824 |
| Leisure activities | 9520 | 2.62 (±2.03) | 2.75 (±1.91) | 1.449 | 0.134 |
| Poor dietary diversity score | 4868 | 353 (46.75) | 4515 (51.54) | 6.357 | 0.012 |
| **Health status** |  |  |  |  |  |
| Normal body mass index (18.5–24.9, kg/m2) | 5398 | 417 (59.32) | 4981 (59.13) | 4.264 | 0.234 |
| Depression | 1200 | 83 (6.92) | 1117 (93.08) | 3.504 | 0.173 |
| Hypertension | 2695 | 226 (29.89) | 2469 (28.17) | 1.047 | 0.593 |
| Diabetes | 393 | 30 (7.63) | 363 (92.37) | 0.251 | 0.882 |
| Heart diseases | 1155 | 90 (11.90) | 1065 (12.15) | 0.053 | 0.974 |
| Stroke | 783 | 81 (10.71) | 702 (8.01) | 6.746 | 0.118 |
| Cancer | 783 | 81 (10.71) | 702 (8.01) | 0.282 | 0.869 |
| Arthritis | 1319 | 103 (13.62) | 1216 (13.87) | 0.471 | 0.79 |
| Cognitive impairment | 2349 | 181 (24.04) | 2168 (24.76) | 0.195 | 0.659 |
| ADL in disability | 2473 | 217 (29.73) | 2256 (26.33) | 3.973 | 0.046 |
| Toothache or pain in jaw joint more than once during the past 6 months | 1654 | 118 (16.39) | 1536 (18.01) | 1.184 | 0.277 |
| **Socioeconomic status** |  |  |  |  |  |
| Poor childhood SES | 1619 | 114 (56.22) | 1505 (67.21) | 65.58 | <0.0001 |
| Poor adult SES | 7800 | 629 (22.49) | 7171 (21.15) | 1.93 | 0.165 |

**Table S2 Association of tourism experiences with mortality in the univariate and multivariable models among 8764 participants who responded at least once to the follow-up 2014 or 2018.**

| Tourism  experiences | Model 1 | |  | Model 2 | |  | Model 3 | |
| --- | --- | --- | --- | --- | --- | --- | --- | --- |
|  | cHR (95% CI) | *P* |  | aHR (95% CI) | *P* |  | aHR (95% CI) | *P* |
| 0 times within the past two years | 1 (reference) |  |  | 1 (reference) |  |  | 1 (reference) |  |
| ≥1 times within the past two years | 0.39 (0.33, 0.45) | <0.0001 |  | 0.57 (0.49,0.66) | <0.0001 |  | 0.72 (0.62, 0.85) | <0.0001 |

Notes: Model 1 was a univariate model. In the model 2, we adjusted basic demographic characteristics, including age, sex, education, residence, marital status and living pattern. In the model 3, we adjusted all the covariates, including age, sex, education, residence, marital status, living pattern smoking status, drinking status, regular exercise, leisure activities, dietary diversity, BMI, depression, cognitive impairment, ADL in disability, history of chronic disease (hypertension, diabetes, heart diseases, stroke, cancer, and arthritis), a toothache or pain in jaw joint more than once during the past 6 months, childhood SES and adult SES. cHR, crude hazard ratio; aHR, adjusted hazard ratio.

**Table S3 Association of tourism experiences with mortality in the multivariable model.**

| Tourism  experiences | Model A^a^ | |  | Model B^b^ | |  |
| --- | --- | --- | --- | --- | --- | --- |
|  | cHR (95% CI) | *P* |  | aHR (95% CI) | *P* |  |
| 0 times within the past two years | 1 (reference) |  |  | 1 (reference) |  |  |
| ≥1 times within the past two years | 0.64 (0.55, 0.75) | <0.0001 |  | 0.74 (0.64,0.86) | <0.0001 |  |

Notes: In the model A, we adjusted all covariates except regular exercise and leisure activities; In the model B, we adjusted all covariates after multiple imputation of missing values; cHR, crude hazard ratio; aHR, adjusted hazard ratio.
